# Epidemiology, outcomes, and prognostic factors in submandibular gland carcinomas: a national DAHANCA study

**DOI:** 10.1007/s00405-023-07940-y

**Published:** 2023-04-13

**Authors:** Marie Westergaard-Nielsen, Christian Godballe, Jesper Grau Eriksen, Stine Rosenkilde Larsen, Katalin Kiss, Tina Agander, Benedicte Parm Ulhøi, Birgitte Wittenborg Charabi, Tejs Ehlers Klug, Henrik Jacobsen, Jørgen Johansen, Claus Andrup Kristensen, Elo Andersen, Maria Andersen, Kristine Bjørndal

**Affiliations:** 1 Department of Otorhinolaryngology, Head and Neck Surgery and Audiology, Odense University Hospital, J.B. Winsloews Vej 4, 5000 Odense C, Denmark; 2 Faculty of Health Sciences, Department of Clinical Research, University of Southern Denmark, Odense, Denmark; 3 Department of Experimental Clinical Oncology, Aarhus University Hospital, Aarhus, Denmark; 4 Department of Pathology, Odense University Hospital, Odense, Denmark; 5 Department of Pathology, Copenhagen University Hospital-Rigshospitalet, Copenhagen, Denmark; 6 Department of Pathology, Aarhus University Hospital, Aarhus, Denmark; 7 Department of Otorhinolaryngology, Head and Neck Surgery, Copenhagen University Hospital-Rigshospitalet, Copenhagen, Denmark; 8 Department of Otorhinolaryngology, Head and Neck Surgery, Aarhus University Hospital, Aarhus, Denmark; 9 Department of Otorhinolaryngology, Head and Neck Surgery, Aalborg University Hospital, Aalborg, Denmark; 10 Department of Oncology, Odense University Hospital, Odense, Denmark; 11 Department of Oncology, Copenhagen University Hospital-Rigshospitalet, Copenhagen, Denmark; 12 Department of Oncology, Copenhagen University Hospital-Herlev and Gentofte, Herlev, Denmark; 13 Department of Oncology, Aalborg University Hospital, Aalborg, Denmark

**Keywords:** Submandibular gland carcinoma, Submandibular gland cancer, Salivary gland carcinoma

## Abstract

**Purpose:**

The aim of this study is to present incidence, histological subtypes, survival rates, and prognostic factors based on a national cohort of patients with salivary gland carcinoma.

**Methods:**

All Danish patients with submandibular gland carcinoma diagnosed from 1990 to 2015 (*n* = 206) were included and analyzed following histological re-evaluation. Data were collected by the Danish Head and Neck Cancer Group (DAHANCA). Overall, disease-specific and recurrence-free survival were evaluated. Prognostic factors were analyzed with multivariate Cox Hazard Regression.

**Results:**

The study population consisted of 109 (53%) men and 97 (47%) women, median age 62 years (range 11–102). Adenoid cystic carcinoma was the most frequent subtype (50%). Tumour classification T1/T2 (75%) and N0 (78%) was most frequent. The mean crude incidence was 0.17/100,000/year. Most patients (*n* = 194, 94%) were treated with primary surgery, and 130 (67%) received postoperative radiotherapy. The 5- and 10-year survival rates were for overall survival 64% and 41%, disease-specific survival 74% and 61%, and recurrence-free survival 70% and 56%, respectively. Survival rates were higher for adenoid cystic carcinoma compared to other subtypes, but the difference was not significant in multivariate analysis. Recurrence occurred in 69 patients, and 37 (53.6%) of them had recurrence in a distant site. Advanced T-classification and regional lymph-node metastases had significant negative impact on survival rates.

**Conclusion:**

The incidence of submandibular gland carcinoma in Denmark was 0.17/100,000/year and stable during the time period. The most frequent subtype was adenoid cystic carcinoma. Half of the recurrences presented in a distant site, and multivariate analysis confirmed that advanced stage was independent negative prognostic factor for recurrence and survival.

**Supplementary Information:**

The online version contains supplementary material available at 10.1007/s00405-023-07940-y.

## Introduction

Salivary gland carcinomas are uncommon and account for only 3–5% of head and neck cancers [1–3]. Although most frequently located in the parotid gland, 5–15% of the salivary gland carcinomas [1, 4–7] are located in the submandibular gland. The proportion of tumours with malignancy varies with the salivary gland from which a tumour origin. Malignancy seems to be more frequent in submandibular gland tumours (22–50%) than in tumours of the parotid gland (9–32%) [1, 6, 8–10], and carcinomas in the submandibular gland have been associated with a higher frequency of high-grade malignancies and poorer prognosis than parotid gland carcinomas [11–13].

Submandibular gland carcinomas consist of a wide variety of histopathological subtypes with different biological behaviours, malignant potentials, and prognoses.

The primary treatment modality is surgery with resection of the submandibular gland with or without neck dissection. Radiotherapy is often offered as adjuvant treatment to the primary tumour site and regional lymph nodes, thus enhancing locoregional control [12, 14, 15]. According to Danish national guidelines, radiotherapy is applied in case of high-grade histological subtype, involved or close surgical margins, regional lymph-node metastases, advanced T-classification, perineural growth, and recurrence [16].

The combination of a low incidence rate and the variability in biological behaviour among the subtypes makes it difficult to evaluate treatment strategies, survival rates, and prognostic factors and requires long term follow-up. In this paper, we present the results from a national study of an unselected complete national cohort of patients with submandibular gland carcinoma during a period of 26 years. The aim is to present results on incidence, survival, and prognostic factors for patients with submandibular gland carcinoma.

## Materials and methods

All patients diagnosed with submandibular gland carcinoma in Denmark between 1st January 1990 and 31st December 2015 were included in this retrospective study. Patients were identified from the Danish Head and Neck Cancer Group (DAHANCA) database, which contains extensive data on all Danish patients with salivary gland carcinoma [4]. A total of 206 patients were diagnosed with primary submandibular gland carcinoma in the inclusion period.

All available histological specimens were histologically re-evaluated by experienced salivary gland pathologists (SRL, KK, TA, and BPU) as part of a previous study [4]. Re-evaluation was performed according to the World Health Organization (WHO) 2005 classification system for patients diagnosed with submandibular gland carcinoma before 1st January 2006, and according to the WHO 2017 classification system for patients diagnosed with submandibular gland carcinoma after this date. Mucoepidermoid carcinomas, adenocarcinomas NOS, and squamous cell carcinomas were graded as high, intermediate, or low grade. Adenoid cystic carcinomas were graded according to their growth patterns (i.e., solid or tubulo-cribriform), both defined as high risk and treated as high-grade carcinomas. Histological subtypes were categorised as high- or low-grade subtypes according to the Danish National Guidelines [16]. All tumours with high-grade pathological features were categorised as high-grade, regardless of histological subtype. The definition of high- and low-grade histological subtypes is provided in Supplementary Table A.

Surgical margins of 5 mm or more were defined as clear.

Tumours were staged according to 8th edition of UICC TNM classification of Malignant Tumours [17].

Patient and tumour characteristics, treatment details, and follow-up data were extracted from the DAHANCA database, medical records, and pathology reports. To ensure any recurrence after the end of regular clinical follow-up was registered, a search in The Danish Pathology Data Bank, which contains all the histological and cytological pathology records in Denmark, was conducted for all patients included in the study.

If data on status at follow-up were not available, date of death and cause of death were extracted from the national Danish Central Person Register and the Cause of Death Register.

### Statistical analysis

Incidence rates were calculated based on population numbers from “Statistics Denmark” [18] and analyzed with Poisson regression. Age-adjusted incidences were calculated according to the WHO World Standard Population 2000–2025 [19]. Estimates of overall survival (OS), recurrence-free survival (RFS), and disease-specific survival (DSS) were calculated by the Kaplan–Meier method. Follow-up time was calculated from the date of first surgical treatment to the date of death or the end of data collection (January 2018). For patients not treated with surgery, the follow-up time was calculated from the date of fine needle aspiration biopsy (FNAB) to the date of death or end of data collection.

Variables with possible association to survival were analyzed with multivariate Cox Proportional Hazard regression. Age, sex, tumour stage (T1/T2 versus T3/T4), nodal status (N0 versus N +), histology (low- versus high-grade subtypes), surgical margins (clear versus involved/close margins), and perineural and vascular invasion (yes or no) were considered as variables. Two-sided *p* values < 0.05 were considered significant. Data were stored in a RedCap database provided by the Open Patient data Explorative Network (OPEN). Statistical analysis was performed with Stata 16 (StataCorp LP, College Station, Texas).

## Results

The study cohort consisted of 206 patients, 109 men and 97 women, with a median age of 62 years (range 11–102). Only two patients were younger than 18 years. The mean crude incidence was 0.17/100,000/year. There was no increase in the incidence during the study period.

Most patients presented with tumour classification T1 or T2 (*n* = 155, 75.2%), and 47 patients (22.8%) had T3/T4 tumours. In four patients, the T-classification was not assessed. The majority, 160 patients (77.7%), had no cervical lymph-node metastases, whereas 45 patients (21.8%) had regional lymph-node metastases. One patient was classified as Nx. N-classification was based on histological evaluation after either neck dissection or selective lymph-node excision in 122 patients (59.2%), and clinical evaluation including diagnostic imaging in 84 patients (40.8%).

Fifteen patients (7.3%) presented with distant metastases at the time of diagnosis.

Adenoid cystic carcinoma was the most frequent subtype (*n* = 103, 50.0%) followed by adenocarcinoma NOS (*n* = 23, 11.2%), carcinoma ex pleomorphic adenoma (*n* = 17, 8.2%), and mucoepidermoid carcinoma (*n* = 16, 7.8%). There were two (1.0%) cases of secretory carcinoma, which was added as a separate subtype in the WHO 2017 classification, and these cases were, therefore, only present in specimens from 2006 to 2015.

Curatively intended surgery was performed in 194 patients (94.1%). In 101 patients (49.0%), the primary surgery also included neck dissection, and in 21 patients (10.8%), a selective lymph-node excision was performed during primary surgery. Adjuvant radiotherapy was given to 137 patients (66.5%).

In eight patients (3.9%), surgery was not favourable, and these patients were treated with primary radiotherapy. The diagnosis was confirmed by a core biopsy from primary tumour and, in one patient, also a lymph-node excision.

Four patients (2.0%) had distant metastases or high stage disease and received palliative radiotherapy or supportive care.

Patient, tumour characteristics, and treatment data are summarized in Table 1.

**Table 1:** Patient and tumour characteristics

A: Characteristics	No. 206	
**Sex**	**n**	**%**
Male	109	53
Female	97	47
Male:female ratio 1:1		
**Age, years**		
Median age (range)	62 (11–102)	
**Pathology**	**n**	**%**
Adenoid cystic carcinoma	103	50
Mucoepidermoid carcinoma	16	7.8
Acinic cell carcinoma	2	1
Adenocarcinoma NOS	23	11.2
Carcinoma ex pleomorphic adenoma	17	8.2
Squamous cell carcinoma	12	5.8
Epithelial-myoepithelial carcinoma	6	2.9
Basal cell adenocarcinoma	2	1
Undifferentiated carcinoma	5	2.4
Myoepithelial carcinoma	2	1
Secretory carcinoma	2	1
Salivary duct carcinoma	10	4.8
Poorly differentiated carcinoma	3	1.4
Other	3	1.4
**Histological grade**	**n**	**%**
Low grade	121	58.7
High grade	85	41.3
**T-classification**	**n**	**%**
T1/T2	155	75.2
T3/T4	47	22.8
TX	4	2
**N-classification**	**n**	**%**
N0	160	77.7
N +	45	21.8
NX	1	0.5
**M-classification**	**n**	**%**
M0	191	92.7
M1	15	7.3
**Stage**	**n**	**%**
I	76	36.9
II	52	25.2
III	30	14.5
IVa	29	14.1
IVb	2	1
IVc	15	7.3
Unknown	2	1
**Treatment**	**n**	**%**
Surgery	57	27.6
Surgery and adjuvant radiotherapy	137	66.5
Primary radiotherapy (with biopsy from primary tumour)	8	3.9
Palliative treatment	4	2
**Surgical margins**	**n**	**%**
Clear	59	28.6
Close	44	21.4
Involved	94	45.6
Unknown	9	4.4

The pathology report described clear surgical margins in 59 (28.6%) patients and involved or close surgical margins in 138 (67.0%) of the patients treated with curatively intended surgery. In nine cases (4.4%), the surgical margins were not assessed in the pathology reports. Perineural invasion was observed in 89 (43.2%) of the cases, and most often in adenoid cystic carcinoma (62/103, 60.2%). It was described as absent in 56 (27.2%) cases, and in 61 (29.6%) cases, this variable was not available in the pathology reports. Correspondingly, vascular invasion was observed in 29 (14.1%), and it was described as absent in 81 (39.3%) cases and was not available in 96 (46.6%) cases.

The median follow-up time for all patients was 5.1 years (range 0.1–28.8 years). For patients alive at the end of follow-up, median follow-up time was 9.3 years (range 2.1–28.8 years).

The 5-year, 10-year, and 15-year OS rates were 64%, 41%, and 38%. Corresponding RFS rates were 70%, 56%, and 53%, and DSS rates were 74%, 61%, and 61%, respectively. There was no statistically significant difference between survival rates for patients with adenoid cystic carcinoma compared to other subtypes. The survival rates for all patients and for patients with adenoid cystic carcinoma are summarized in Table 2.

**Table 2:** Survival rates, A: all included patients, B: patients with adenoid cystic carcinoma (ACC)

A: Survival rates, all patients, *n* = 206	5-year (95% CI)	10-year (95% CI)	15-year (95% CI)
Overall survival	64 (57; 70)	41 (33; 48)	38 (30; 46)
Recurrence-free survival	70 (63; 76)	56 (47; 63)	53 (45; 61)
Disease-specific survival	74 (67; 79)	61 (53; 69)	61 (53; 69)
Number at risk after the period	108	51	36

B: Survival rates, patients with ACC, *n* = 103	5-year (95% CI)	10-year (95% CI)	15-year (95% CI)
Overall survival	79 (69; 86)	53 (41; 64)	49 (37; 60)
Recurrence-free survival	80 (70; 87)	64 (52; 74)	60 (47; 70)
Disease-specific survival	83 (74; 89)	69 (57; 78)	69 (57; 78)
Number at risk after the period	65	32	22

*CI* confidence interval

**Fig. 1:**
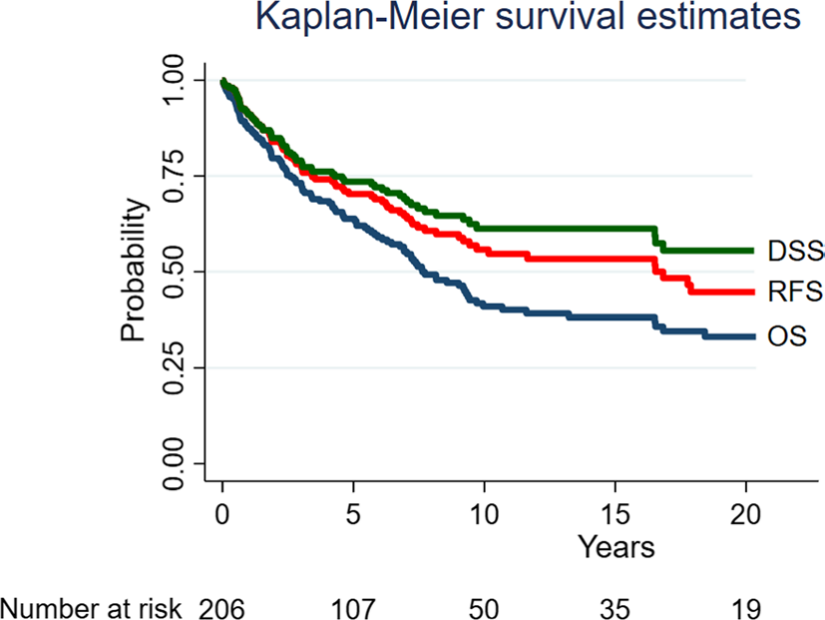
Survival rates illustrated with Kaplan–Meier curves. *OS* overall survival, *RFS* recurrence-free survival, *DSS* disease-specific survival

**Fig. 2:**
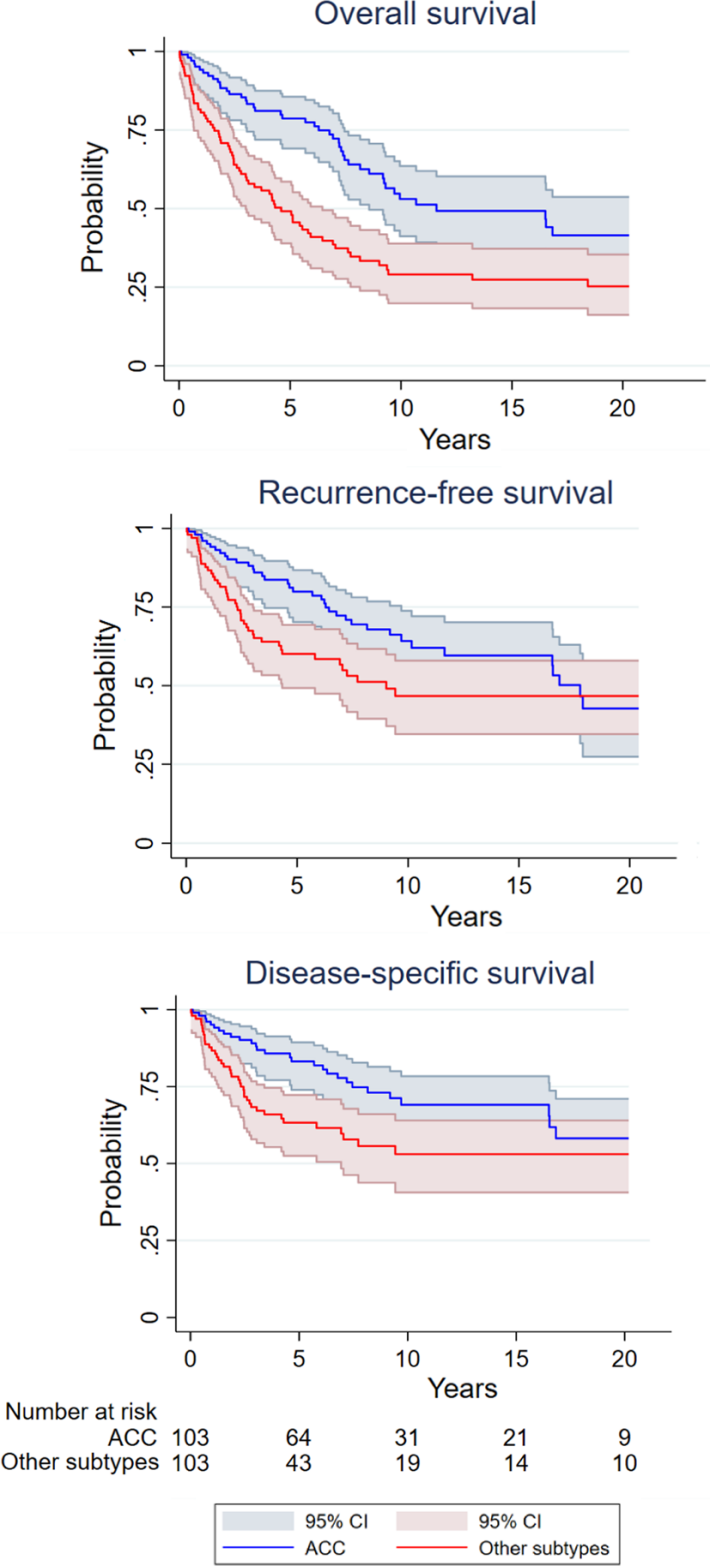
Survival rates stratified by patients with adenoid cystic carcinoma and patients with other histological subtypes

Kaplan–Meier plots of overall survival (OS), disease-specific survival (DSS), and recurrence-free survival (RFS) are shown in Fig. 1. Kaplan–Meier plots stratified by adenoid cystic carcinoma and other subtypes are shown in Fig. 2.

In the multivariate Cox Proportional Hazard regression analysis, advanced T-classification (T3/T4) and cervical lymph-node metastases (N +) had significant negative impact on both DSS and RFS. Vascular invasion was negatively associated with OS, and age above 60 years were negatively associated with both OS and RFS. Involved/close surgical margins had significant negative impact on RFS. Hazard ratios (HR) are summarized in Table 3. Multivariate analyses of the patients with adenoid cystic carcinoma showed that the presence of perineural invasion had a significant negative impact on RFS (HR 2.02, *p* = 0.044), but no significant influence on OS or DSS.

**Table 3:** Multivariate analyses on factors associated with survival rates

	Overall survival		Disease-specific survival		Recurrence-free survival	
	Hazard ratio (95% CI)	*p* value	Hazard ratio (95% CI)	*p* value	Hazard ratio (95% CI)	*p* value
Age > 60 years	**3.1 (2.0, 4.8)**	** < *0.001***	1.7 (1.0, 2.9)	*0.071*	**1.7 (1.0, 2.8)**	***0.033***
Male gender	1.3 (0.8, 2.0)	*0.248*	1.2 (0.7, 2.2)	*0.442*	1.2 (0.7, 2.0)	*0.431*
High-grade histology	1.3 (0.8, 2.3)	*0.335*	2.1 (0.9, 4.8)	*0.067*	1.6 (0.8, 3.2)	*0.152*
T3/T4	1.5 (0.9,2.4)	*0.121*	**2.6 (1.4, 4.6)**	***0.002***	**2.4 (1.4, 4.1)**	***0.002***
N +	**3.1 (2.0,4.9)**	** < *0.001***	**4.4 (2.5, 7.9)**	** < *0.001***	**4.4 (2.6, 7.5)**	** < *0.001***
Involved/close surgical margins	1.2 (0.8, 2.0)	*0.397*	1.9 (0.9, 3.8)	*0.071*	**1.8 (1.0, 3.2)**	***0.050***
Perineural invasion	1.3 (0.8, 2.5)	*0.393*	1.5 (0.7, 3.3)	*0.353*	1.5 (0.7, 2.9)	*0.278*
Vascular invasion	**2.3 (1.2, 4.5)**	***0.009***	2.2 (0.9, 5.2)	*0.078*	2.1 (1.0, 4.4)	*0.061*

Significant values are noted with bold text

Italic indicates two-sided* p* values < 0.05 were considered significant

**Fig. 3:**
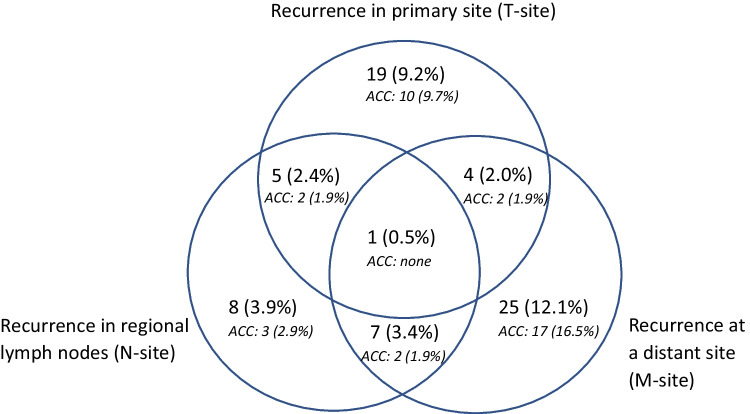
Recurrence pattern. Recurrence during follow-up period: *n* = 69. Patients with adenoid cystic carcinoma (ACC) noted below the number of patients in the total cohort

Recurrence occurred in 69 patients treated with curative intent (69/194, 35.6%). The recurrence pattern is shown in Fig. 3. The recurrence rate was highest in patients with salivary duct carcinoma (6/10, 60.0%) and adenocarcinoma (10/23, 43.5%). Patients with adenoid cystic carcinoma presented with recurrence in tumour site in 14 cases (14/103, 13.6%), regional lymph nodes in 7 cases (7/103, 6.8%), and distant site in 21 cases (21/103, 20.4%). Recurrence rates divided by histological subtype are summarized in Table 4.

**Table 4:** Recurrence and disease-specific survival (DSS) according to histological subtypes

Histological subtype	No. of patients	Recurrence	Recurrence site *(%)*			DSS	
		No. *(%)*	T	N	M	5-Year (95% CI)	10-Year (95% CI)
Adenoid cystic carcinoma	103	36 *(35.0)*	14 *(13.6)*	7 *(6.8)*	21 *(20.4)*	83 (74; 89)	69 (57; 78)
Mucoepidermoid carcinoma	16	4 *(25.0)*	2 *(12.5)*	1 (6.3)	1 (6.3)	75 (46; 90)	75 (46; 90
Acinic cell carcinoma	2	0	–	–	–	100	100
Adenocarcinoma NOS	23	10 *(43.5)*	5 *(21.7)*	6 *(26.0)*	7 *(30.4)*	42 (20; 63)	42 (20; 63)
Carcinoma ex pleo. adenoma	17	6 *(35.3)*	5 *(29.4)*	2 *(11.8)*	1 *(6.9)*	74 (45; 90)	50 (16; 77)
Squamous cell carcinoma	12	5 *(41.7)*	3 *(25.0)*	2 *(16.7)*	1 *(8.3)*	55 (23; 78)	0
Epithelial-myoepithelial carcinoma	6	0	–	–	–	100	100
Basal cell adenocarcinoma	2	0	–	–	–	0	0
Undifferentiated carcinoma	5	0	–	–	–	80 (20; 97)	80 (20; 97)
Myoepithelial carcinoma	2	0	–	–	–	100	100
Secretory carcinoma	2	0	–	–	–	0	0
Salivary duct carcinoma	10	6 *(60.0)*	0	2 *(20.0)*	4 *(40.0)*	0	0
Poorly differentiated carcinoma	3	1 *(33.3)*	0	0	1 *(33.3)*	0	0
Other	3	1 *(33.3)*	0	1 *(33.3)*	1 *(33.3)*	0	0
Total	206	69 *(34.0)*	29 *(14.1)*	21 *(10.2)*	37 *(18.0)*	74 (67; 79)	61 (53; 69)

Percentage noted with italics

Median time to recurrence was 2.0 years (range 0.2–21.5 years). Eighteen patients (18/69, 26.1%) with recurrence were treated for recurrent disease and had no evidence of disease at further follow-up, 36 patients (36/69, 52.2%) were treated for recurrence, but had active disease at follow-up, and 15 patients (15/69, 21.7%) had untreated recurrence. Fifteen patients had recurrence more than 5 years after the primary treatment. Most of these patients (13/15, 86.7%) had adenoid cystic carcinoma and two patients had mucoepidermoid and carcinoma ex pleomorphic adenoma, respectively. Recurrence after 10 years or more occurred in six patients with adenoid cystic carcinoma and one patient with carcinoma ex pleomorphic adenoma.

At the end of follow-up, 91 patients (44.2%) were alive. Death was caused by submandibular gland carcinoma in 67 patients (58.3%), other cancers in 18 patients (15.6%), and other diseases in 23 patients (20.0%). Four patients (3.5%) died during treatment and three patients (2.6%) died from unknown causes.

## Discussion

Submandibular gland carcinoma is uncommon, and the rare occurrence was confirmed in this retrospective study of all patients diagnosed with salivary gland carcinoma in Denmark in a period of 26 years. During this period, in average, eight patients were diagnosed with submandibular carcinoma each year, which corresponded a crude incidence of 0.17/100,000 per year in Denmark. Comparison of national incidence rates is challenging, because the majority of studies are based on small cohorts from a single or multiple institutions, but results from a large study by Lee et al. [20], based on the SEER cohort, and studies from the Nordic countries [5, 8, 21], have shown similar low incidence rates.

Consistent with other studies on submandibular gland carcinoma, the most frequent subtype was adenoid cystic carcinoma [5, 20–26]. In this cohort, half of the patients had adenoid cystic carcinoma. Others have reported a proportion of adenoid cystic carcinoma between 29 and 57% in patients with submandibular gland carcinoma [5, 20–26]. This representation of histological subtypes is different from parotid gland carcinoma, where acinic cell and mucoepidermoid carcinoma are more common [2, 4, 7, 27]. More than half (58.7%) of the patients had high-grade histological subtypes. This has also been reported in several similar studies [23, 26], whereas others have reported lower proportions (28–44%) of patients with high-grade histological carcinoma [20–22].

Most studies have reported 5-year OS between 50 and 60% [15, 20, 22, 26] consistent our results, whereas others have shown higher rates with 5-year OS 72–77% [23, 28–30]. Those that have reported 10-year OS, show rates similar to ours with 10-year OS between 36 and 40% [15, 20, 30].

The 5-year RFS for all patients in this study was found to be 70% and may reflect that the majority of the patients had T1 or T2 tumours. Lower rates have been reported (46–57%) [15, 22, 26], whereas others have found 5-year RFS similar to our result (66–72%) [25, 28, 30]. A few studies have reported 10-year RFS, but these results were similar to ours, with 10-year RFS 52–66% [15, 30].

We found that DSS may be relatively stable after 10 years follow-up. Comparable studies have reported 5-year DSS with a wide range between 53 and 76% [15, 20–22, 25, 26, 30]. Similar to this study, Lee et al. [20] reported the 10-year DSS to be 60%, whereas other studies have reported lower rates with 10-year DSS 41–51% [15, 30].

The differences among DSS reported in studies on submandibular gland carcinoma may be explained by differences in patient population and the proportion of histological subtypes within the study cohorts. In this study, we showed a difference in DSS between the four most common histological subtypes. The 5-year DSS for patients with adenoid cystic carcinoma was twice the rate for patients with adenocarcinoma NOS. The 5-year DSS was similar for patients with mucoepidermoid carcinoma and carcinoma ex pleomorphic adenoma, but both lower than DSS for patients with adenoid cystic carcinoma.

In contrast to earlier studies [4], histological grade did not have significant influence on the survival rates in this study. This is likely to be explained by differences in treatment based on histological grade, and patients with high-grade histological tumours received postoperative radiotherapy. Negative impact of histological grade on DSS has been reported in other studies of patients with submandibular gland carcinoma [20, 22, 23, 26]. Skalova et al. [31] reported that salivary gland carcinomas with high-grade transformation have a higher risk of recurrence, higher risk of regional metastases and a poorer prognosis than low-grade carcinomas. High-grade transformation has been described in several histological subtypes including some of the subtypes, that are commonly considered low grade [31]. There was no significant difference in recurrence rates among the individual histological subtypes in this study. This result may also have been influenced by the fact that patients with high-grade histological subtype received postoperative radiotherapy and the treatment differed for the subtypes. However, patients with involved/close surgical margins were also treated with postoperative radiotherapy, and this had significant negative impact on the RFS independently of histological grade. For patients with ACC, perineural invasion also increased the risk of recurrence (e.g., had negative impact on RFS), and these patients were treated with postoperative radiotherapy based on both histological subtype and perineural invasion.

The histological grade or subtype is often unknown prior to surgery, because the preoperative diagnosis is based on fine needle aspiration biopsy (FNAB). Though FNAB can identify malignancies from benign lesions in salivary gland neoplasms [32], the cytological classification of histological subtype or grade is inaccurate [33, 34]. The surgical treatment consists of excision of the submandibular gland, but the inclusion and extent of neck dissection may depend on the histological grade in case of no clinically suspected regional metastases.

Tumour size or T-classification as well as regional metastases (e.g., stage) has also proved to have a significant negative impact on survival rates [15, 20, 25]. The relatively high proportion of patients with T1/T2 and N0 disease in this study may have influenced the survival rates. Three-fourths (75.2%) of the patients had T1/T2 tumours and 77.7% of the patients had no regional metastases, which lead to a relatively high proportion of patients with disease stage 1 and 2 (36.9% and 25.3%, respectively). The proportion of patients with regional metastases (N +) was lower (22%) than reported by comparable studies, in which 34–50% of the patients presented with regional metastases [22, 23, 25, 26, 35, 36]. A few patients (7.3%) presented with distant metastases, but more than half of the patients with recurrence (54%) had recurrence in a distant site. High frequencies of distant recurrence have also been reported in other studies of submandibular gland carcinoma [22, 23, 26, 37, 38], implying that even in patients with sufficient surgical and radiotherapeutic treatment, there is a considerable risk of systematic failure. An increasing number of studies on the effect of chemotherapy, targeted therapy, and immunotherapy on salivary gland carcinoma have been performed [39], but the prognosis for patients with distant metastases is poor.

Though associated with a fair prognosis and survival for up to 5 to 10 years of follow-up [12, 22], adenoid cystic carcinoma has also been characterised by late development of distant metastases [15, 37, 40, 41]. Our results confirmed this characteristic, as almost only patients with ACC presented with recurrences more than 10 years after primary diagnosis. In this study, 20% of the patients with adenoid cystic carcinoma developed distant metastases, while only 7% developed regional metastases. This difference between the proportion of recurrences in regional lymph nodes and in a distant site in patients with adenoid cystic carcinoma has also been reported by others [37].

The strengths of this study are inclusion of a complete national cohort of unselected patients with submandibular gland carcinoma with a follow-up period of up to 25 years. The histological specimens were re-evaluated by pathologists specialized in salivary gland pathology to ensure the histological diagnosis. The re-evaluation of histological specimens from 1990 to 2005 was performed according to the WHO classification from 2005 [6], whereas specimens from 2006 to 2015 were re-evaluated and classified according to the WHO classification from 2017 [42]. Though this may have affected the proportion of histological subtypes between the two periods, the most common subtypes, adenoid cystic carcinoma, mucoepidermoid carcinoma, adenocarcinoma NOS, and carcinoma ex pleomorphic adenoma persisted unchanged through the WHO classification editions including the most recent 5^th^ edition from 2022 [43].

As all retrospective studies, this study was limited by the restrictions in data collection from medical records and surgical reports. Data on perineural and vascular invasion were missing in a high proportion of the pathology reports. These variables were not available despite histological re-evaluation, because the re-evaluation was performed with focus on diagnosing the histological subtype and did not include reviewing histological slides of the whole specimens. The retrospective design also complicates the interpretation of radiotherapy as a variable with possible impact on outcome. Patients with high-risk disease were selected for radiotherapy, and only in patients with low risk (e.g. low-grade histological tumours with no suspected lymph-node metastases and radical surgery), radiotherapy was opted out.

## Conclusion

The present study contributes further knowledge on the epidemiology, prognostic factors, and outcome for patients with submandibular gland carcinoma. We reported a low overall incidence rate, with adenoid cystic carcinoma as the most frequent subtype. The survival rates varied slightly according to histological subtypes, but DSS was relatively stable after 10-year follow-up in the total cohort. Despite curatively intended treatment, almost a fifth of the patients had recurrence with distant metastases. We found that advanced T-classification and regional metastases had significantly negative impact on prognosis.

## Supplementary Information

Below is the link to the electronic supplementary material.

Supplementary file1 (DOCX 22 KB)

## Data Availability

The data that support the findings of this study are available from the corresponding author upon reasonable request.
