# Supplementary material for: Epidemiology, outcomes, and prognostic factors in submandibular gland carcinomas: a national DAHANCA study
Source: Eur Arch Otorhinolaryngol. 2023 Apr 13;280(7):3405–13. doi: 10.1007/s00405-023-07940-y (PMC10219881; doi:10.1007/s00405-023-07940-y)
Supplement: Supplementary file 1 — Supplementary file1 (DOCX 22 KB) [file 405_2023_7940_MOESM1_ESM.docx]

Supplementary Table A, Definition of high- and low risk histological subtypes. All tumours with high grade transformation are defined as high grade

| **LOW RISK** | **HIGH RISK** |
| --- | --- |
| Mucoepidermoid carcinoma  (high and intermediate differentiation) | Mucoepidermoid carcinoma  (low differentiation) |
| Acinic cell carcinoma | Adenoid cystic carcinoma (solid and tubulocribriform) |
| Adenocarcinoma NOS(high differentiation) | Adenocarcinoma NOS (intermediate and low diff.) |
| Carcinoma ex pleomorphic adenoma  (Intracapsular/minimally invasive or with low grade histology) | Carcinoma ex pleomporphic adenoma  (widely invasive or high grade histology) |
| Epithelial-myoepithelial carcinoma | Sebaceous adenocarcinoma |
| Polymorphous adenocarcinoma | Squamous cell carcinoma |
| Secretory carcinoma | Carcinosarcoma |
| Clear cell carcinoma | Lymphoepithelial carcinoma |
| Basal cell carcinoma | Salivary duct carcinoma |
| Intraductal carcinoma | Myoepithelial carcinoma |
|  | Poorly differentiated carcinoma  Undifferentiated carcinoma  Large cell neuroendocrine carcinoma  Small cell neuroendocrine carcinoma |
|  | (Oncocytic carcinoma) |
